# Halogen Bonding versus Hydrogen Bonding: A Molecular Orbital Perspective

**DOI:** 10.1002/open.201100015

**Published:** 2012-04-04

**Authors:** Lando P Wolters, F Matthias Bickelhaupt

**Affiliations:** [a] Department of Theoretical Chemistry, Amsterdam Center for Multiscale Modeling, Vrije Universiteit AmsterdamDe Boelelaan 1083, 1081 HV Amsterdam (The Netherlands) E-mail: F.M.Bickelhaupt@vu.nl

**Keywords:** activation strain models, bond theories, density functional calculations, halogen bonds, hydrogen bonds

## Abstract

We have carried out extensive computational analyses of the structure and bonding mechanism in trihalides DX⋅⋅⋅A^−^ and the analogous hydrogen-bonded complexes DH⋅⋅⋅A^−^ (D, X, A=F, Cl, Br, I) using relativistic density functional theory (DFT) at zeroth-order regular approximation ZORA-BP86/TZ2P. One purpose was to obtain a set of consistent data from which reliable trends in structure and stability can be inferred over a large range of systems. The main objective was to achieve a detailed understanding of the nature of halogen bonds, how they resemble, and also how they differ from, the better understood hydrogen bonds. Thus, we present an accurate physical model of the halogen bond based on quantitative Kohn–Sham molecular orbital (MO) theory, energy decomposition analyses (EDA) and Voronoi deformation density (VDD) analyses of the charge distribution. It appears that the halogen bond in DX⋅⋅⋅A^−^ arises not only from classical electrostatic attraction but also receives substantial stabilization from HOMO–LUMO interactions between the lone pair of A^−^ and the σ* orbital of D–X.

## Introduction

Hydrogen bonds are, without doubt, one of the most important intermolecular interactions known today. Being responsible for the unique features of water, as well as playing a key role in DNA structure and replication, the importance of hydrogen bonding for human life can hardly be overestimated. Therefore, it is no surprise that this interaction has been studied extensively.[1, 2] Halogen bonds, although discovered around 150 years ago,[3] have received considerably less attention. After the first experimental studies on this phenomenon by Hassel,[4] a review by Bent appeared in which donor–acceptor interactions with halogens were discussed.[5] Early theoretical descriptions were given by Pimentel,[6] Mulliken,[7] and Flurry.[8] In the last decades, there has been an increased interest in halogen bonding, which nowadays has applications in various fields in chemistry,[9] such as, supramolecular,[10–12] biochemistry[13–15] and inorganic chemistry.[16] Earlier studies generally indicate that halogen bonds can, both in terms of practical applications and bond strength, compete with hydrogen bonds.[17–24]

In this study, we have computationally investigated a range of strongly halogen-bonded trihalides DX⋅⋅⋅A^−^ and the analogous strongly hydrogen-bonded complexes DH⋅⋅⋅A^−^ (D, X, A=F, Cl, Br, I), using relativistic density functional theory (DFT). The purpose of our work is twofold. Firstly, we wish to provide a set of consistent structural and energy data from which reliable trends can be inferred for a wide range of model systems. The second and main objective is to achieve a detailed understanding of the nature of halogen bonds: how they resemble, and also how they differ from, the better understood hydrogen bonds in terms of their electronic structure and bonding mechanism.

To this end, we first explore how the geometries and energies of our model complexes DX⋅⋅⋅A^−^ and, for comparison, DH⋅⋅⋅A^−^ vary as either the halogen- or hydrogen-bond donating atom (D), or the halogen- or hydrogen-bond accepting atom (A) is varied from F to Cl, Br and I. In this way, we arrive at a set of consistent data for a large range of halogen- and hydrogen-bonded complexes. Next, to understand the origin of the computed trends, we carry out activation strain analyses[25] of the bond formation reaction. The activation strain model of chemical reactivity[25] is a fragment-based approach to understand the energy profile of a chemical process in terms of the original reactants: the strain energy associated with their geometrical deformation and their mutual interaction along the reaction coordinate (see below). The interaction energy and the underlying bonding mechanism are furthermore analyzed in the context of quantitative Kohn–Sham molecular orbital (MO) theory in combination with an energy decomposition analysis (EDA).[26, 27] Our explorations and analyses augment earlier pioneering studies[28–31] through the large variety in our halogen- and hydrogen-bonded model complexes and the systematic and in-depth analyses along the entire reaction profile for each of the complexation reactions.

## Theoretical Methods

### Computational details

All calculations were carried out using the Amsterdam density functional (ADF) program developed by Baerends and co-workers.[32–34] The numerical integration was performed using the procedure developed by te Velde et al.[35] The MOs were expanded in a large uncontracted set of Slater-type orbitals (STOs) containing diffuse functions, TZ2P (no Gaussian functions are involved). The TZ2P basis set[36] is of triple-ζ quality for all atoms and has been augmented with two sets of polarization functions, that is, 2p and 3d on H, 3d and 4f on F and Cl, 4d and 4f on Br, and 5d and 4f on I. The core shells of the halogen atoms (1s for F, 1s2s2p for Cl, up to 3p for Br and up to 4p for I) were treated by the frozen-core approximation. An auxiliary set of s, p, d, f and g STOs was used to fit the molecular density and to represent the Coulomb and exchange potentials accurately in each self-consistent field (SCF) cycle.

Equilibrium structures were obtained by optimizations using analytical gradient techniques.[37] Geometries and energies were calculated at the BP86 level of the generalized gradient approximation (GGA); exchange is described by Slater's Xα potential,[38] with nonlocal corrections due to Becke[39] added self-consistently, and correlation is treated in the Vosko-Wilk-Nusair (VWN) parameterization[40] with nonlocal corrections due to Perdew[41] added, again, self-consistently (BP86).[42] Scalar relativistic effects were accounted for using the zeroth-order regular approximation (ZORA).[43] Energy minima have been verified to be equilibrium structures through vibrational analysis.[44] All minima were found to have zero imaginary frequencies.

Throughout this paper, we focus on the electronic energies of the molecular systems. However, enthalpies at 298.15 K and 1 atm are calculated using standard statistical mechanics relationships as well.[45] The thermodynamic effects were found to have only a small influence on the energies and do not alter the trends. For clarity, these results are, therefore, not discussed but are included in the Supporting Information.

### Analysis of the bonding mechanism

Insight into the bonding mechanism is obtained through activation strain analyses of the various hydrogen- and halogen-bond formation reactions. These complexation reactions are computationally modeled by decreasing the distance between A^−^ and the DH or DX fragment, and simultaneously increasing the D–H or D–X bond length. The DH⋅⋅⋅A^−^ or DX⋅⋅⋅A^−^ distance is decreased from an initial value of 1.8 times[46] the equilibrium bond length in the corresponding HA or XA molecule to the actual bond length value in the hydrogen- or halogen-bonded complex (*r*_H⋅⋅⋅A^−^_ or *r*_X⋅⋅⋅A^−^_), while the DH or DX fragment is stretched from its equilibrium geometry to the geometry it acquires in the hydrogen- or halogen-bonded complex. Thus, each analysis starts from an optimized DH or DX molecule and a halide at a relatively large distance, which is then linearly transformed to the optimized hydrogen- or halogen-bonded complex.

These complexation reactions are analyzed using the activation strain model. The activation strain model of chemical reactivity[25] is a fragment-based approach to understand the energy profile of a chemical process in terms of the original reactants. Thus, the potential energy surface Δ*E*(ζ) is decomposed along the reaction coordinate ζ (or just at one point along ζ) into the strain energy Δ*E*_strain_(ζ), which is associated with the geometrical deformation of the individual reactants as the process takes place, plus the actual interaction energy Δ*E*_int_(ζ) between the deformed reactants [Eq. (1)].


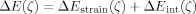
(1)

In the equilibrium geometry, that is, for ζ = ζ_eq_, this yields an expression for the bond energy Δ*E*(ζ_eq_)=Δ*E*=Δ*E*_strain_ + Δ*E*_int_. The PyFrag program was used to facilitate the analyses along the reaction coordinate ζ of the bond formation processes.[47] The interaction energy Δ*E*_int_(ζ) between the deformed reactants is further analyzed in the conceptual framework provided by the quantitative Kohn–Sham MO model.[26] To this end, it is decomposed in three physically meaningful terms [Eq. (2)] using a quantitative energy decomposition scheme developed by Ziegler and Rauk.[27]


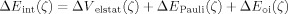
(2)

The term Δ*V*_elstat_ corresponds to the classical Coulomb interaction between the unperturbed charge distributions of the deformed reactants and is usually attractive. The Pauli repulsion energy (Δ*E*_Pauli_) comprises the destabilizing interactions between occupied orbitals of the reactants and is responsible for steric repulsion. The orbital-interaction energy (Δ*E*_oi_) accounts for charge transfer, that is, the interaction between occupied orbitals of one fragment with unoccupied orbitals of the other fragment, including the interactions of the highest occupied and lowest unoccupied MOs (HOMO–LUMO), and polarization, that is, empty–occupied orbital mixing on one fragment, due to the presence of another fragment. Since the Kohn–Sham MO method of DFT in principle yields exact energies, and rather accurate energies in practice, with the available density functionals for exchange and correlation, we have the special situation that an MO method, in principle, completely accounts for the bonding energy.

The electron density distribution is analyzed using the Voronoi deformation density (VDD) method for computing atomic charges.[48] The VDD atomic charge on atom A (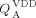) is computed as the (numerical) integral of the deformation density in the volume of the Voronoi cell of atom A [Eq. (3)]. The Voronoi cell of atom A is defined as the compartment of space bounded by the bond midplanes on and perpendicular to all bond axes between nucleus A and its neighboring nuclei.


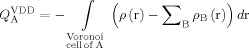
(3)

Here, *ρ*(r) is the electron density of the molecule and ∑_B_*ρ*_B_(r) the superposition of atomic densities *ρ_B_* of a fictitious promolecule without chemical interactions where all atoms are considered neutral. The interpretation of the VDD charge 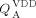 is rather straightforward and transparent: instead of measuring the amount of charge associated with a particular atom A, 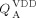 directly monitors how much charge flows out of (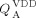
>0) or into (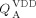
<0) the Voronoi cell of atom A due to chemical interactions.

## Results and Discussion

### Hydrogen-bond strength and structure

The results of our ZORA-BP86/TZ2P calculations are shown in Table 1 for a representative selection of hydrogen-, fluorine- and iodine-bonded model complexes DX⋅⋅⋅A^−^, covering D=F and I, X=H, F and I, as well as A^−^=F^−^, Cl^−^, Br^−^ and I^−^ (full data on all model systems can be found in Tables S1–S10 in the Supporting Information). In the first place, we note that all model reactions are associated with single-well potential energy surfaces (PES), that is, there is no separate energy minima for DX⋅⋅⋅A^−^ and D⋅⋅⋅XA^−^. In the case where D=A, *D*_∞h_ symmetric complexes with equal bond distances *r*_D–X_=*r*_X⋅⋅⋅A^−^_ are formed (see Table 1). Furthermore, bond enthalpies at 298 K (Δ*H*^298^) show the same trends as the electronic bond energies Δ*E* with differences in the order of one kcal mol^−1^ or less (see Tables S1–S5 in the Supporting Information). Thus, for clarity and a straightforward connection with our activation strain analyses, our discussion is based on the trends in electronic bond energies Δ*E*.

**Table 1:** Bond lengths [Å] and energies relative to reactants [kcal mol^−1^] of the hydrogen-, fluorine- and iodine-bonded complexes[a]

DX⋅⋅⋅A^−^	*r*_D–X_	Δ*r*_D–X_[b]	*r*_X⋅⋅⋅A^−^_	Δ*r*_X⋅⋅⋅A^−^_[c]	Δ*E*	Δ*E*_strain_	Δ*E*_int_	BDE_D–X_[d]
FH⋅⋅⋅F^−^	1.159	0.226	1.159	0.226	−53.0	19.7	−72.8	143.5
FH⋅⋅⋅Cl^−^	1.012	0.079	1.843	0.550	−26.6	3.3	−29.8	143.5
FH⋅⋅⋅Br^−^	0.994	0.061	2.058	0.625	−21.9	2.0	−23.9	143.5
FH⋅⋅⋅I^−^	0.982	0.049	2.319	0.694	−18.1	1.3	−19.4	143.5
IH⋅⋅⋅F^−^	2.319	0.694	0.982	0.049	−80.6	40.9	−121.4	81.7
IH⋅⋅⋅Cl^−^	2.191	0.566	1.423	0.130	−38.0	31.7	−69.6	81.7
IH⋅⋅⋅Br^−^	2.057	0.432	1.642	0.209	−28.6	21.8	−50.3	81.7
IH⋅⋅⋅I^−^	1.941	0.316	1.941	0.316	−21.8	13.6	−35.4	81.7
FF⋅⋅⋅F^−^	1.755	0.335	1.755	0.335	−51.5	23.5	−75.0	50.1
FF⋅⋅⋅Cl^−^	1.864	0.444	1.965	0.301	−43.3	34.2	−77.5	50.1
FF⋅⋅⋅Br^−^	1.902	0.482	2.049	0.253	−44.0	37.7	−81.7	50.1
FF⋅⋅⋅I^−^	1.993	0.573	2.126	0.181	−48.4	46.0	−94.3	50.1
IF⋅⋅⋅F^−^	2.126	0.181	1.993	0.573	−23.9	5.9	−29.8	75.3
IF⋅⋅⋅Cl^−^	2.158	0.213	2.294	0.630	−14.5	7.8	−22.3	75.3
IF⋅⋅⋅Br^−^	2.200	0.255	2.335	0.539	−14.5	10.5	−25.0	75.3
IF⋅⋅⋅I^−^	2.324	0.379	2.324	0.379	−16.9	19.3	−36.2	75.3
FI⋅⋅⋅F^−^	2.129	0.184	2.129	0.184	−75.0	6.1	−81.1	75.3
FI⋅⋅⋅Cl^−^	2.124	0.179	2.620	0.268	−49.8	5.8	−55.6	75.3
FI⋅⋅⋅Br^−^	2.126	0.181	2.781	0.275	−45.1	5.9	−51.0	75.3
FI⋅⋅⋅I^−^	2.132	0.187	2.977	0.277	−41.9	6.3	−48.2	75.3
II⋅⋅⋅F^−^	2.977	0.277	2.132	0.187	−69.0	6.4	−75.4	49.0
II⋅⋅⋅Cl^−^	2.971	0.271	2.632	0.280	−44.2	6.1	−50.3	49.0
II⋅⋅⋅Br^−^	2.976	0.276	2.795	0.289	−39.9	6.4	−46.3	49.0
II⋅⋅⋅I^−^	2.991	0.291	2.991	0.291	−37.4	6.9	−44.3	49.0

[a] Computed at ZORA-BP86/TZ2P.

[b] Stretch of the D–X fragment relative to the optimized DX molecule.

[c] Change in X–A distance compared to the situation in the optimized XA molecule.

[d] BDE_D–X_ is the homolytic bond dissociation energy of the D–X bond without ZPE [kcal mol^−1^]

For the hydrogen-bonded DH⋅⋅⋅A^−^ complexes, we find that as we vary the hydrogen-bond accepting halide A^−^ from F^−^ to I^−^, the hydrogen-bond strength is weakened, *r*_H⋅⋅⋅A^−^_ becomes longer, and the D–H bond becomes less elongated from its equilibrium value in an isolated DH molecule (Δ*r*_D–H_= *r*_D–HA^−^_−*r*_D–H_). The opposite trend emerges as we vary the hydrogen-bond donating atom D in DH down group 17 (F^−^ to I^−^). Thus, along the hydrogen halides from FH to IH, the hydrogen-bond strength is reinforced, the *r*_H⋅⋅⋅A^−^_ bond length becomes smaller, and the bond stretch Δ*r*_D–H_ increases.

For example, from FH⋅⋅⋅F^−^ to FH⋅⋅⋅I^−^, the hydrogen-bond strength (Δ*E*) is weakened from −53 to −18 kcal mol^−1^, while the *r*_H⋅⋅⋅A^−^_ value increases from 1.159 to 2.319 Å, and the value of the bond stretch Δ*r*_D–H_ is reduced from 0.226 to 0.049 Å (see Table 1). This trend correlates with a systematic weakening of the halide's proton affinity (PA) value of 373 kcal mol^−1^ for F^−^ to 316 kcal mol^−1^ for I^−^ (PA values at ZORA-BP86/QZ4P taken from Ref. [49]). The effect is even more pronounced in the series from IH⋅⋅⋅F^−^ to IH⋅⋅⋅I^−^ along which Δ*E* weakens from a value of −81 to −22 kcal mol^−1^, the *r*_H⋅⋅⋅A^−^_ value increases from 0.982 to 1.941 Å, and the Δ*r*_D–H_ value is reduced from 0.694 to 0.316 Å. Note that, on the other hand, from FH⋅⋅⋅F^−^ to IH⋅⋅⋅F^−^, Δ*E* is strengthened from a value of −53 to −81 kcal mol^−1^, while the value of *r*_H⋅⋅⋅A^−^_ decreases from 1.159 to 0.982 Å, and the stretch value Δ*r*_D–H_ is increased from 0.226 to 0.694 Å. The higher extent of deformation in the more strongly hydrogen-bonded complexes is also reflected by a more destabilizing strain energy (Δ*E*_strain_; see Table 1). This trend correlates with a systematic weakening of the halogen–hydrogen bond from a homolytic bond dissociation energy (BDE) value of 144 kcal mol^−1^ in FH to 82 kcal mol^−1^ in IH (see Table 2). Furthermore, note that the bond distance *r*_H⋅⋅⋅A^−^_ in DH⋅⋅⋅A^−^ is in all cases longer than that of *r*_H–A_ in the diatomic HA molecule, as revealed by the corresponding difference in bond distances Δ*r*_H⋅⋅⋅A^−^_=*r*_H⋅⋅⋅A^−^_−*r*_H–A_. This difference Δ*r*_H⋅⋅⋅A^−^_ increases in value from 0.226 Å in FH⋅⋅⋅F^−^ to 0.694 Å in FH⋅⋅⋅I^−^ and from 0.049 Å in IH⋅⋅⋅F^−^ to 0.316 Å in IH⋅⋅⋅I^−^.

**Table 2:** Geometry, stability, and electronic structure of DH and DX molecules[a]

D–X	*r*_D–X_	BDE	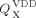	*ε*(σ)	*ε*(σ^*^)	*ε*(*π*)	*ε*(π^*^)
F–H	0.933	143.5	0.20	−13.57	−0.72	−9.78	–
Cl–H	1.293	107.5	0.10	−11.79	−0.97	−8.05	–
Br–H	1.433	94.6	0.07	−11.18	−1.42	−7.51	–
I–H	1.625	81.7	0.05	−10.31	−1.88	−6.91	–
F–F	1.420	50.1	0.00	−15.61	−6.17	−13.05	−9.74
Cl–F	1.664	69.2	−0.07	−13.61	−4.86	−11.66	−8.04
Br–F	1.796	69.8	−0.11	−12.86	−5.04	−11.01	−7.63
I–F	1.945	75.3	−0.13	−11.95	−4.86	−10.49	−7.03
F–Cl	1.664	69.2	0.07	−13.61	−4.86	−11.66	−8.04
Cl–Cl	2.023	62.0	0.00	−11.93	−4.51	−9.89	−7.37
Br–Cl	2.173	58.8	−0.03	−11.38	−4.71	−9.36	−7.13
I–Cl	2.352	57.9	−0.08	−10.72	−4.67	−8.93	−6.78
F–Br	1.796	69.8	0.11	−12.86	−5.04	−11.01	−7.63
Cl–Br	2.173	58.8	0.03	−11.38	−4.71	−9.36	−7.13
Br–Br	2.321	55.0	0.00	−10.88	−4.82	−8.86	−6.93
I–Br	2.506	53.0	−0.06	−10.26	−4.73	−8.41	−6.62
F–I	1.945	75.3	0.13	−11.95	−4.86	−10.49	−7.03
Cl–I	2.352	57.9	0.08	−10.72	−4.67	−8.93	−6.78
Br–I	2.506	53.0	0.06	−10.26	−4.73	−8.41	−6.62
I–I	2.700	49.0	0.00	−9.68	−4.65	−7.92	−6.39

[a] Computed at ZORA-BP86/TZ2P; *r*_D−X_=D–X distance [Å]; BDE=homolytic bond dissociation energy without ZPE [kcal mol^−1^]; 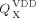
=VDD charge on atom X [au]; *ε*=orbital energy [eV].

We conclude that the DH⋅⋅⋅A^−^ hydrogen bond becomes stronger and relatively shorter, while the D–H bond becomes more elongated in the complex, as the A^−^ anion is a stronger base and/or the D–H bond is weaker.

### Halogen-bond strength and structure

In part, the halogen bonds display similar trends to the hydrogen bonds, but there are also striking differences. In general and in agreement with ab initio results, the fluorine bonds are the weakest and the iodine bonds the strongest halogen bonds.[21, 23, 24] The heavier DX⋅⋅⋅A^−^ halogen bonds (i.e., X=Cl, Br and I) become weaker and longer as the accepting halide (A^−^) varies from F^−^ to I^−^, similar to the corresponding hydrogen bonds. In the case of the iodine-bonded complexes DI⋅⋅⋅A^−^, for example, Δ*E* weakens from a value of around −70 kcal mol^−1^ for A^−^=F^−^ to around −40 kcal mol^−1^ for X^−^=I^−^ (see Table 1). However, the fluorine bonds DF⋅⋅⋅A^−^ display a more complex dependency of Δ*E* upon variation of the accepting halide A^−^. From A^−^=F^−^ to Cl^−^, the fluorine bond strength still weakens, similar to the situation for the hydrogen bonds and the heavier halogen bonds. However, thereafter, along A^−^=Cl^−^, Br^−^ and I^−^, the fluorine-bond strength no longer continues to weaken but instead becomes stronger. This is most clearly seen in the series constituted by the complex FF⋅⋅⋅A^−^ between a fluorine molecule and a halide ion. Here, Δ*E* for the fluorine-bond strength varies along A^−^=F^−^, Cl^−^, Br^−^ and I^−^ with values of −52, −43, −44 and −48 kcal mol^−1^, respectively (see Table 1).

Interestingly, variation of the donating atom D has opposite effects on the halogen bonds DX⋅⋅⋅A^−^ and hydrogen bonds DH⋅⋅⋅A^−^. All halogen bonds studied here become weaker and longer as D is varied from F to I (see Table 1), whereas the hydrogen bonds were found to become stronger and shorter along this series (see above). For example, along the series from FF⋅⋅⋅F^−^ to IF⋅⋅⋅F^−^, the fluorine-bond strength weakens from a Δ*E* value of −52 to only −24 kcal mol^−1^, the fluorine-bond distance *r*_X⋅⋅⋅A^−^_ increases in value from 1.755 to 1.993 Å, and the stretch Δ*r*_D–X_ decreases in value from 0.335 to 0.181 Å.

### Bond analyses with variation of A^−^

**Figure 1:**
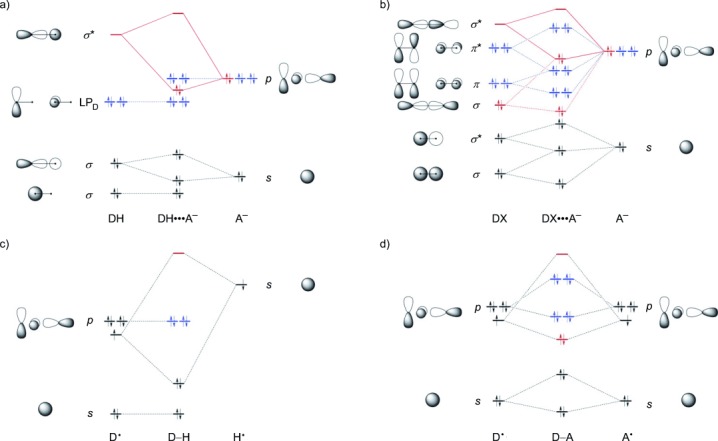
Simplified orbital-interaction diagrams for a) hydrogen-bonded complexes DH⋅⋅⋅A^−^, b) halogen-bonded complexes DX⋅⋅⋅A^−^, c) hydrogen halides D–H, and d) dihalogens D–X, as they emerge from our quantitative Kohn–Sham MO analyses.

Our analyses show that the weakening of hydrogen bonds DH⋅⋅⋅A^−^ and of heavier halogen bonds DX⋅⋅⋅A^−^ (X=Cl, Br, I), as the accepting group varies from A^−^=F^−^ to I^−^, is directly related to the concomitant reduction in electron-donating capacity of the np-type HOMO of the A^−^ halide. The hydrogen and halogen bonds appear to have an electrostatic component (Δ*V*_elstat_) and a covalent component (Δ*E*_oi_) stemming mainly from the HOMO–LUMO interaction between the occupied halide np atomic orbital (AO) and the D–H or D–X antibonding σ* acceptor orbital, shown schematically in Figure 1. Both bonding components, Δ*V*_elstat_ and Δ*E*_oi_, are weakened as the halide HOMO becomes more diffuse and effectively lower in energy[50] from A^−^=F^−^ to I^−^ (see Tables S6, S8–S10 in the Supporting Information). Consequently, the interaction energy (Δ*E*_int_) and, thus, the net hydrogen- or halogen-bond strength Δ*E* becomes less stabilizing along A^−^=F^−^ to I^−^ (see Table 1 and Tables S1 and S3–S5 in the Supporting Information).

**Figure 2:**
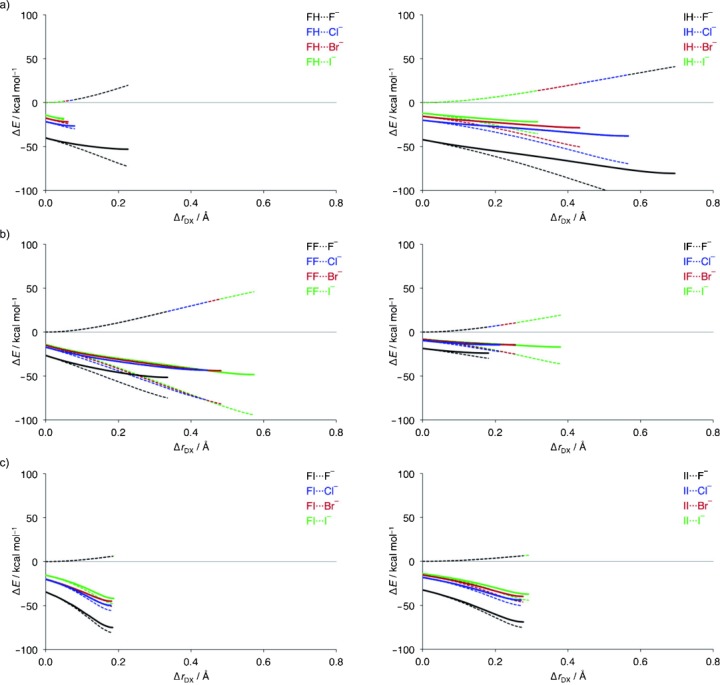
Activation strain analyses along the reaction coordinate for DX + A^−^ complexation as a function of A^−^=F^−^, Cl^−^, Br^−^ and I^−^, projected onto the D–X stretch Δ*r*_DX_ for a) hydrogen bonds, b) fluorine bonds and c) iodine bonds, with donating groups D=F (left) and D=I (right). Energy profiles Δ*E* (solid lines) are decomposed into strain energy Δ*E*_strain_ (dashed lines above Δ*E*=0) and interaction energy Δ*E*_int_ (dashed lines below Δ*E*=0).

The key to understanding why fluorine bonds DF⋅⋅⋅A^−^ show a more complex, partially opposite trend (i.e., the expected weakening from A^−^=F^−^ to Cl^−^ but thereafter a strengthening along A^−^=Cl^−^, Br^−^ and I^−^) is contained in the counteracting effects evolving from D–F bond stretching induced in the diatomic DF molecule as it interacts with the halide A^−^. Interestingly, activation strain analyses reveal that from an early until a relatively advanced stage of the complexation reaction, for a given point along the reaction coordinate ζ, we indeed recover the original trend in interactions, namely, that Δ*E*_int_(ζ) weakens from A^−^=F^−^ to I^−^. This can be nicely seen in Figure 2 which, for six representative series, shows the activation strain diagrams along the entire reaction coordinate ζ projected onto the stretch Δ*r*_D–X_ of the complexation reaction between a DX molecule approaching the halogen-bond accepting A^−^ (see section, Theoretical Methods). Each of the six activation strain diagrams in Figure 2 refers to one particular DH or DX molecule forming a hydrogen or halogen bond with A^−^=F^−^, Cl^−^, Br^−^ and I^−^. The strain-energy curves (Δ*E*_strain_) within each of these subgraphs coincide because they refer to the same diatomic molecule being stretched as the complexation reaction progresses. Consequently, the trend A^−^=F^−^ to I^−^ in the total DH⋅⋅⋅A^−^ and DX⋅⋅⋅A^−^ energy profiles Δ*E* in each subgraph is directly determined by the trend in the corresponding interaction-energy curves (Δ*E*_int_). Also, as can be seen in Figure 2, the Δ*E*_int_ curve appears to be most stabilizing for A^−^=F^−^ and then weakens along Cl^−^, Br^−^ and I^−^, for any given diatomic molecule DH or DX, including all fluorine-bonded molecules DF.

In other words, fluorine bonds DF⋅⋅⋅A^−^ would also show a weakening in interaction Δ*E*_int_ from A^−^=F^−^ to I^−^, as the hydrogen bonds and all other halogen bonds, if it were not for the increasingly stretched D–F bond in the fluorine-bond-donating diatomic molecule (see Table 1 and Figure 2). This structural phenomenon is promoted by a combination of factors: 1) a weak D–X bond that is easily stretched; 2) a strong interaction with an approaching halide A^−^; and importantly, 3) a DX σ* acceptor orbital that quickly drops in energy as the D–X bond elongates (see Figure 1). The latter generates a driving force for D–X stretching in DX⋅⋅⋅A^−^ because it enhances the orbital interactions and thus Δ*E*_int_ (see Figures 1 and 2). Indeed, D–X stretching is most pronounced if this bond in the diatomic fragment is weaker, that is, for the weaker halogen–hydrogen bonds (D–X=I–H) and the weaker halogen–halogen bonds (D–X=F–F; see Table 1). In the latter, it is able to affect the trend in overall bond strength Δ*E*. The D–F stretching in fluorine-bonded complexes is most pronounced in the FF⋅⋅⋅A^−^ series, along which the F–F stretch Δ*r*_D–X_ increases from a value of 0.3 via 0.4 and 0.5 to 0.6 Å. This further stretch is able to induce the reversal of the trend in bond strength Δ*E* along the equilibrium structures FF⋅⋅⋅Cl^−^, FF⋅⋅⋅Br^−^ and FF⋅⋅⋅I^−^ (see Table 1).

Thus, fluorine-bond analyses in the DF⋅⋅⋅A^−^ equilibrium geometries show that in most cases the interaction energy (Δ*E*_int_) between the stretched D–F molecule and the halide A^−^, as well as its components Δ*V*_elstat_ and Δ*E*_oi_, become more stabilizing along the entire series A^−^=F^−^ to I^−^, that is, already from F^−^ to Cl^−^ (see Table 1, S2 and S7 in the Supporting Information). This is indeed most pronouncedly so in the series FF⋅⋅⋅A^−^, due to the F–F bond in the DX fragment being relatively weak. Along the series FF⋅⋅⋅F^−^, FF⋅⋅⋅Cl^−^, FF⋅⋅⋅Br^−^ and FF⋅⋅⋅I^−^, Δ*E*_int_ increases in strength from a value of −75 to −78, −82, and −94 kcal mol^−1^, respectively. For comparison, along the corresponding series with the much stronger F–I bond in the DX fragment, that is, FI⋅⋅⋅F^−^, FI⋅⋅⋅Cl^−^, FI⋅⋅⋅Br^−^ and FI⋅⋅⋅I^−^, the Δ*E*_int_ weakens from −81 to −56, −51, and −48 kcal mol^−1^.

The overall bond strength Δ*E* along the fluorine-bonded series shows the aforementioned initial weakening followed by a strengthening, because the D–F stretching and the concomitant strain energy (Δ*E*_strain_) becomes more destabilizing along the series and, from A^−^=F^−^ to Cl^−^, dominates the strengthening in Δ*E*_int_ (see Table 1).

We conclude that, in general, hydrogen bonds DH⋅⋅⋅A^−^ and halogen bonds DX⋅⋅⋅A^−^ become weaker along A^−^=F^−^ to I^−^ because the larger radii and lower np AO energies of the halides lead to weaker electrostatic attraction and weaker orbital interactions. Interestingly, for the same reason, F^−^ is the halide with the strongest gas-phase basicity, the strongest alkyl cation affinity and the lowest barrier for S_N_2 reactions with halomethanes.[49–51] The trend in DF⋅⋅⋅A^−^ fluorine-bond strength is partially inverted, that is, Δ*E* becomes more stabilizing along A^−^=Cl^−^, Br^−^ and I^−^ because of a more subtle interplay of factors. Notably, a significant stretching of the relatively weak D–F bond in the DF⋅⋅⋅A^−^ equilibrium structures lowers the DF σ* acceptor orbital and thus amplifies the donor–acceptor orbital interactions, for example, along FF⋅⋅⋅Cl^−^, FF⋅⋅⋅Br^−^ and FF⋅⋅⋅I^−^.

### Bond analyses with variation of D

**Figure 3:**
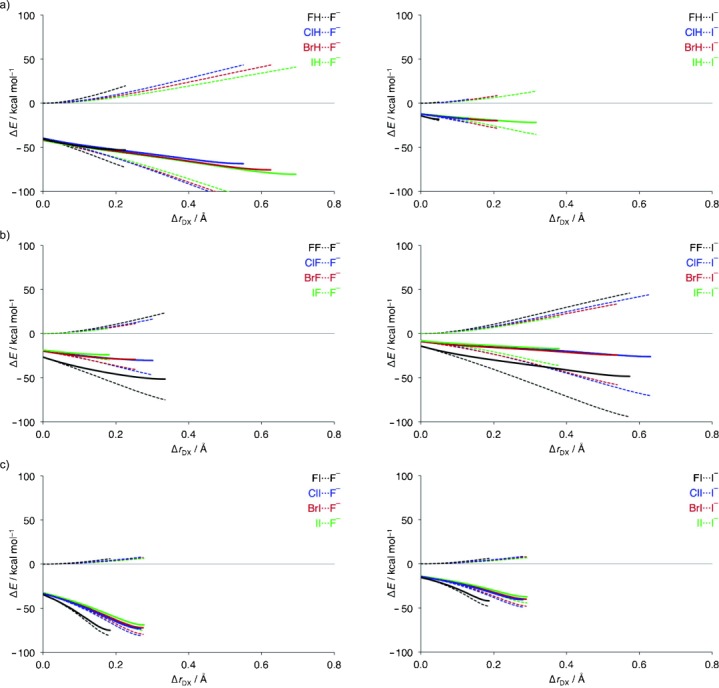
Activation strain analyses along the reaction coordinate for DX + A^−^ complexation as function of D=F, Cl, Br and I, projected onto the D–X stretch Δ*r*_DX_ for a) hydrogen bonds, b) fluorine bonds and c) iodine bonds, with accepting groups A^−^=F^−^ (left) and A^−^=I^−^ (right). Energy profiles Δ*E* (solid lines) are decomposed into strain energy Δ*E*_strain_ (dashed lines above Δ*E*=0) and interaction energy Δ*E*_int_ (dashed lines below Δ*E*=0).

We recall that for the hydrogen bonds DH⋅⋅⋅A^−^, a heavier donating halogen D results in a stronger bond, whereas the same variation in D weakens the halogen bonds DX⋅⋅⋅A^−^ (see Table 1 and S1–S5 in the Supporting Information). In both cases, the trend in bond strength Δ*E* is determined by the interaction energy Δ*E*_int_. For example, from FH⋅⋅⋅F^−^ to IH⋅⋅⋅F^−^, Δ*E*_int_ is strengthened from a value of −71 to −121 kcal mol^−1^, whereas from FI⋅⋅⋅F^−^ to II⋅⋅⋅F^−^ it is weakened from a value of −75 to −29 kcal mol^−1^ (see Table 1). The strain energy (Δ*E*_strain_) is not negligible, but it does not alter the trend set by Δ*E*_int_. Our activation strain analyses explain the above differences between hydrogen and halogen bonds, but they also confirm once more that both are very similar in nature (see Figure 3).

Starting with some general observations, we find that for hydrogen as well as halogen bonds, the strain-energy (Δ*E*_strain_) curves are most unfavorable when D=F and gradually become less destabilizing as the donating atom is varied along D=F, Cl, Br and I (see Figure 3). Furthermore, we find that for all DH⋅⋅⋅A^−^ and DX⋅⋅⋅A^−^ complexes, the interaction-energy (Δ*E*_int_) curves become less stabilizing along D=F, Cl, Br and I. The resulting energy profiles and, therefore, the stability and geometric properties of the complexes DH⋅⋅⋅A^−^ and DX⋅⋅⋅A^−^ depend on the balance between the Δ*E*_strain_ and Δ*E*_int_ curves, which we first discuss individually.

The slope and shape of the Δ*E*_strain_ curves is of course directly related to the D–X bond strength of the diatomic fragment, which in general becomes stronger as the polarity across the D–H or D–X bond increases (see Table 2). This is a well-known and understood phenomenon.[52] From FH to IH, the halogen–hydrogen bond strength decreases significantly from a value of 143 to 82 kcal mol^−1^ (Table 2). The corresponding halogen–halogen bonds are all much weaker, and variations in the homolytic BDE are also much smaller. From FF to IF, the bond strength increases from 50 kcal mol^−1^ to 75 kcal mol^−1^, while for the fragments DX, where X is Cl, Br or I, the bond strength generally decreases from a value of around 70 kcal mol^−1^ for FX to around 50 kcal mol^−1^ for IX. Thus, for the hydrogen-bonded complexes, the Δ*E*_strain_ curves show a pronounced reduction in slope from FH to IH, which, in the corresponding hydrogen-bonded complexes FH⋅⋅⋅A^−^ to IH⋅⋅⋅A^−^, translates into an increasing stretch Δ*r*_D–H_ of the diatomic fragment. As the stretch Δ*r*_D–H_ becomes larger from equilibrium structures FH⋅⋅⋅A^−^ to IH⋅⋅⋅A^−^, the Δ*E*_int_ curves have been able to descend further, to lower, more stabilizing energies. The final result is, thus, an increasing stability of the DH⋅⋅⋅A^−^ complexes when the donating atom D is varied from F to I.

For the halogen bonds, the Δ*E*_strain_ curves are very similar and not decisive. The reason for the decreased stability of the DX⋅⋅⋅A^−^ complexes upon the same variation of D from F to I is, therefore, that the Δ*E*_int_ curves descend more gradually to overall less stabilizing values. Δ*E*_int_ becomes less stabilizing from FX⋅⋅⋅A^−^ to IX⋅⋅⋅A^−^ because of decreasing electrostatic attractions (Δ*V*_elstat_) and, in some cases, also because of greater Pauli repulsions (Δ*E*_Pauli_; see Tables S6–S10 in the Supporting Information). Both of these effects are easily explained considering the electronegativities of the halogens. Along the series FX to IX, the central atom X becomes relatively more electronegative, which will lead to a greater negative charge on this central atom, thus, reducing the electrostatic attraction with the anionic A^−^, while concomitantly the occupied orbitals will have more X character, which in turn introduces stronger Pauli repulsion.

### Bond analyses with variation of X

**Figure 4:**
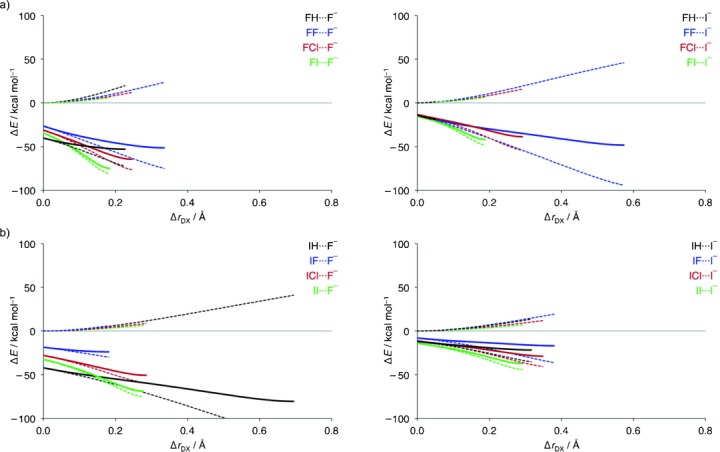
Activation strain analyses along the reaction coordinate for DX + A^−^ complexation as function of X=H, F, Cl and I, projected onto the D–X stretch Δ*r*_DX_ for bond donating groups a) D=F and b) D=I, and accepting groups A^−^=F^−^ (left) and A^−^=I^−^ (right). Energy profiles Δ*E* (solid lines) are decomposed into strain energy Δ*E*_strain_ (dashed lines above Δ*E*=0) and interaction energy Δ*E*_int_ (dashed lines below Δ*E*=0).

A more direct comparison of hydrogen and halogen bonds DX⋅⋅⋅A^−^ can be obtained by varying X along H, F, Cl and I, while keeping the donating atom (D) and the accepting halide (A^−^) constant (see Figure 4). We do this for four combinations of D and A^−^, giving rise to four subgraphs in Figure 4. There appears to be a regular trend of increasing strength from the fluorine bonds to the iodine bonds. This trend derives again directly from the electronegativity difference across the D–X bond of the diatomic fragment; from DF to DI, the charge distribution on the DX fragment is increasingly polarized towards D, away from A^−^ (see VDD atomic charges in Table 2), whereas the σ* acceptor orbital achieves a higher amplitude on X (see Figure 1). This results in a strengthening of the halogen bond DX⋅⋅⋅A^−^ because of greater electrostatic attraction, less Pauli repulsion and more stabilizing donor–acceptor orbital interactions (see Tables S7–S10 in the Supporting Information).

In analogy to the situation described above, hydrogen bonds might be expected to be much stronger than the halogen bonds due to the large and favorable polarization across the D–H bond leading to a partially positively charged hydrogen atom in DH. For example, the VDD atomic charge on X in FH, FF and FI amounts to +0.20, 0.00 and +0.13 au, respectively (see Table 2). The decomposition of the interaction energy into its components shows indeed a stronger electrostatic attraction (Δ*V*_elstat_) to the bonding energy in the case of the hydrogen bonds (compare Tables S6–S10 in the Supporting Information). Note, however, that this does not imply that hydrogen bonds are always stronger than the corresponding halogen bonds, since in our model systems, the bonding mechanism is never purely, or even predominantly, electrostatic. The covalent or orbital-interaction term (Δ*E*_oi_) is relatively large and crucial for understanding the bonding in our model systems. For the hydrogen-bonded complexes DH⋅⋅⋅A^−^, the orbital-interaction term accounts for 40 to 66 % of the total bonding interactions (Δ*V*_elstat_+Δ*E*_oi_). The stabilization due to this term results predominantly from a charge transfer from the np orbitals of the halide into the σ* LUMO of the hydrogen halide (see Figure 1). For the halogen-bonded complexes DX⋅⋅⋅A^−^, the contribution from the orbital-interaction term ranges from 43 % for FI⋅⋅⋅F^−^ to as much as 97 % for IF⋅⋅⋅F^−^ at the other end of the spectrum. The larger covalent contribution, in the case of the halogen bonds, is the result of the low orbital energy of the empty dihalogen σ* orbital (e.g., −0.7 eV for FH and −6.2 eV for FF; see Table 2), which directly translates into a stronger donor–acceptor orbital interaction with the halide np orbital (compare Tables S6–S10 in the Supporting Information). Note that, percentagewise, Δ*E*_oi_ in the halogen bonds appears even larger because of the aforementioned, less favorable electrostatic attraction Δ*V*_elstat_.

The nature of the strong hydrogen and halogen bonds discussed in this work strongly resembles that of the weaker, neutral hydrogen and halogen bonds, although dispersion interactions become relatively more important in the latter.[2, 19, 24, 31] Preliminary results of dispersion-corrected ZORA-BP86-D3/TZ2P calculations on FI⋅⋅⋅FI (Δ*E*=−4.3 kcal mol^−1^), ClCl⋅⋅⋅ClCl (Δ*E*=−1.3 kcal mol^−1^) and II⋅⋅⋅II (Δ*E*=−6.6 kcal mol^−1^) show that the covalent component Δ*E*_oi_ amounts to 43–59 %, whereas dispersion contributes 2–17 % to the total of all bonding interactions (Δ*E*_oi_ + Δ*V*_elstat_ +dispersion interaction; see Theoretical Methods and ref. [53] for the D3-dispersion correction as proposed by Grimme). The covalent contribution in these neutral model complexes stems from a donor–acceptor orbital interaction from an occupied π* orbital on one dihalogen fragment into the σ* orbital of the other dihalogen fragment.

We conclude that halogen bonds DH⋅⋅⋅A^−^ and hydrogen bonds DX⋅⋅⋅A^−^ have a very similar bonding mechanism consisting of both electrostatic and covalent contributions. The electrostatic attraction is less favorable in the halogen bonds due to a smaller and in some cases less favorably oriented polarization across the dihalogen molecule DX. Nevertheless, halogen bonds can become stronger than hydrogen bonds because of a more stabilizing covalent component in the former. The reason is the lower orbital energy of the empty σ* orbitals in dihalogen molecules DX leading to a stronger, more favorable donor–acceptor orbital interaction with the halide A^−^ np orbital (see Table 2).

## Conclusion

Halogen bonds in DX⋅⋅⋅A^−^ are very similar in nature to hydrogen bonds in DH⋅⋅⋅A^−^ (D, X, A=F, Cl, Br, I): both have a sizeable covalent component stemming from HOMO–LUMO interactions between the np-type lone pair on the halogen- or hydrogen-bond accepting fragment A^−^ and the D–X or D–H antibonding σ* LUMO on the halogen- or hydrogen-bond donating fragment DX or DH, respectively. Neither halogen bonds nor hydrogen bonds are, therefore, predominantly, let alone purely electrostatic phenomena. This follows from our bonding analyses based on relativistic density functional theory (DFT).

Two characteristic differences between the halogen bonds DX⋅⋅⋅A^−^ and hydrogen bonds DH⋅⋅⋅A^−^ is that the former are generally associated with a weaker electrostatic attraction (dihalogens DX are less polar than hydrogen halides DH) and a significantly more stabilizing HOMO–LUMO interaction. The stronger orbital interaction derives from the lower energy of the halogen–halogen σ* LUMO as compared with that of the much stronger halogen–hydrogen bond. Halogen bonds can be stronger but also weaker than the corresponding hydrogen bonds.

Finally, hydrogen bonds DH⋅⋅⋅A^−^ and halogen bonds DX⋅⋅⋅A^−^ become weaker along A^−^=F^−^ to I^−^, because the electron-donating capability (and basicity, alkyl cation affinity, nucleophilicity)[49, 51] of the halide decreases in this order. The trend in DF⋅⋅⋅A^−^ fluorine-bond strength is partially inverted, that is, Δ*E* becomes more stabilizing along A^−^=Cl^−^, Br^−^ and I^−^, because of a more subtle interplay of factors, in which a significant stretching of the relatively weak D–F bond lowers the DF σ* acceptor orbital and, thus, amplifies the donor–acceptor orbital interactions.

## Supplementary material

Detailed facts of importance to specialist readers are published as ”Supporting Information”. Such documents are peer-reviewed, but not copy-edited or typeset. They are made available as submitted by the authors.
