# Supplementary material for: Halogen Bonding versus Hydrogen Bonding: A Molecular Orbital Perspective
Source: ChemistryOpen. 2012 Apr 4;1(2):96–105. doi: 10.1002/open.201100015 (PMC3922460; doi:10.1002/open.201100015)
Supplement: Supplementary file 1 [file open0001-0096-SD1.pdf]

ChemistryOPEN

Supporting Information

© Copyright Wiley-VCH Verlag GmbH & Co. KGaA, 69451 Weinheim, 2012

Halogen Bonding versus Hydrogen Bonding: A Molecular Orbital Perspective

Lando P. Wolters and F. Matthias Bickelhaupt*[a]

open_201100015_sm_miscellaneous_information.pdf

Content

Table S1. Bond lengths and energies relative to reactants of hydrogen-bonded complexes.

Table S2. Bond lengths and energies relative to reactants of fluorine-bonded complexes.

Table S3. Bond lengths and energies relative to reactants of chlorine-bonded complexes.

Table S4. Bond lengths and energies relative to reactants of bromine-bonded complexes.

Table S5. Bond lengths and energies relative to reactants of iodine-bonded complexes.

Table S6. Analysis of hydrogen-bonding mechanism in $DH\cdots A^-$ complexes.

Table S7. Analysis of fluorine-bonding mechanism in $DF\cdots A^-$ complexes.

Table S8. Analysis of chlorine-bonding mechanism in $DCl\cdots A^-$ complexes.

Table S9. Analysis of bromine-bonding mechanism in $DBr\cdots A^-$ complexes.

Table S10. Analysis of iodine-bonding mechanism in $DI\cdots A^-$ complexes.

Table S1. Bond lengths (in Å) and energies (in kcal mol^{-1}) relative to reactants of hydrogen-bonded complexes.[a]

DX···A⁻	$r_{D\text{-}H}$	$\Delta r_{D\text{-}H}$[b]	$r_{H\cdots A^-}$	$\Delta r_{H\cdots A^-}$[c]	ΔH^{298}	ΔE	ΔE_{strain}	ΔE_{int}
FH···F⁻	1.159	0.226	1.159	0.226	–53.4	–53.0	19.7	–72.8
FH···Cl⁻	1.012	0.079	1.843	0.550	–26.7	–26.6	3.3	–29.8
FH···Br⁻	0.994	0.061	2.058	0.625	–21.9	–21.9	2.0	–23.9
FH···I⁻	0.982	0.049	2.319	0.694	–18.1	–18.1	1.3	–19.4
ClH···F⁻	1.843	0.550	1.012	0.079	–67.1	–68.6	43.3	–111.9
ClH···Cl⁻	1.587	0.294	1.587	0.294	–30.3	–29.3	17.9	–47.2
ClH···Br⁻	1.478	0.185	1.874	0.441	–23.4	–22.4	8.4	–30.9
ClH···I⁻	1.423	0.130	2.191	0.566	–18.2	–17.5	4.6	–22.1
BrH···F⁻	2.058	0.625	0.994	0.061	–73.6	–75.6	43.2	–118.8
BrH···Cl⁻	1.874	0.441	1.478	0.185	–34.6	–34.1	27.4	–61.5
BrH···Br⁻	1.743	0.310	1.743	0.310	–26.7	–25.7	15.6	–41.3
BrH···I⁻	1.642	0.209	2.057	0.432	–20.8	–19.8	8.6	–28.4
IH···F⁻	2.319	0.694	0.982	0.049	–78.1	–80.6	40.9	–121.4
IH···Cl⁻	2.191	0.566	1.423	0.130	–37.8	–38.0	31.7	–69.6
IH···Br⁻	2.057	0.432	1.642	0.209	–29.1	–28.6	21.8	–50.3
IH···I⁻	1.941	0.316	1.941	0.316	–22.7	–21.8	13.6	–35.4

[a] Computed at ZORA-BP86/TZ2P. [b] D–H stretch relative to optimized DH molecule. [b] H···A⁻ distance relative to optimized HA molecule.

Table S2. Bond lengths (in Å) and energies (in kcal mol^{-1}) relative to reactants of fluorine-bonded complexes.[a]

DX···A$^-$	$r_{D\text{-}X}$	$\Delta r_{D\text{-}X}$ [b]	$r_{X\cdots A^-}$	$\Delta r_{X\cdots A^-}$ [c]	ΔH^{298}	ΔE	ΔE_{strain}	ΔE_{int}
FF···F$^-$	1.755	0.335	1.755	0.335	–51.5	–51.5	23.5	–75.0
FF···Cl$^-$	1.864	0.444	1.965	0.301	–43.5	–43.3	34.2	–77.5
FF···Br$^-$	1.902	0.482	2.049	0.253	–44.2	–44.0	37.7	–81.7
FF···I$^-$	1.993	0.573	2.126	0.181	–48.7	–48.4	46.0	–94.3
ClF···F$^-$	1.965	0.301	1.864	0.444	–30.2	–30.3	16.4	–46.7
ClF···Cl$^-$	2.077	0.413	2.077	0.413	–21.2	–21.2	26.1	–47.3
ClF···Br$^-$	2.143	0.479	2.126	0.330	–21.9	–21.8	31.8	–53.6
ClF···I$^-$	2.294	0.630	2.158	0.213	–26.1	–26.0	44.4	–70.4
BrF···F$^-$	2.049	0.253	1.902	0.482	–29.0	–29.2	11.4	–40.6
BrF···Cl$^-$	2.126	0.330	2.143	0.479	–20.0	–20.0	17.2	–37.2
BrF···Br$^-$	2.186	0.390	2.186	0.390	–20.4	–20.5	21.9	–42.3
BrF···I$^-$	2.335	0.539	2.200	0.255	–24.2	–24.2	33.7	–57.9
IF···F$^-$	2.126	0.181	1.993	0.573	–23.7	–23.9	5.9	–29.8
IF···Cl$^-$	2.158	0.213	2.294	0.630	–14.4	–14.5	7.8	–22.3
IF···Br$^-$	2.200	0.255	2.335	0.539	–14.4	–14.5	10.5	–25.0
IF···I$^-$	2.324	0.379	2.324	0.379	–16.9	–16.9	19.3	–36.2

[a] Computed at ZORA-BP86/TZ2P. [b] D–X stretch relative to optimized DX molecule. [c] X···A$^-$ distance relative to optimized XA molecule.

Table S3. Bond lengths (in Å) and energies (in kcal mol^{-1}) relative to reactants of chlorine-bonded complexes.[a]

DX···A$^-$	$r_{D\text{-}X}$	$\Delta r_{D\text{-}X}$[b]	$r_{X\cdots A^-}$	$\Delta r_{X\cdots A^-}$[c]	ΔH^{298}	ΔE	ΔE_{strain}	ΔE_{int}
FCl···F$^-$	1.909	0.245	1.909	0.245	–64.2	–64.5	11.9	–76.4
FCl···Cl$^-$	1.925	0.261	2.334	0.311	–43.8	–43.9	13.2	–57.1
FCl···Br$^-$	1.933	0.269	2.473	0.300	–40.4	–40.5	13.8	–54.3
FCl···I$^-$	1.955	0.291	2.637	0.285	–38.7	–38.7	15.6	–54.3
ClCl···F$^-$	2.334	0.311	1.925	0.261	–56.8	–57.2	12.8	–70.0
ClCl···Cl$^-$	2.354	0.331	2.354	0.331	–37.3	–37.5	14.1	–51.6
ClCl···Br$^-$	2.366	0.343	2.495	0.322	–34.3	–34.5	14.8	–49.3
ClCl···I$^-$	2.399	0.376	2.654	0.302	–33.3	–33.4	17.1	–50.5
BrCl···F$^-$	2.473	0.300	1.933	0.269	–55.3	–55.8	10.9	–66.6
BrCl···Cl$^-$	2.495	0.322	2.366	0.343	–36.2	–36.5	12.1	–48.6
BrCl···Br$^-$	2.507	0.334	2.507	0.334	–33.4	–33.7	12.8	–46.4
BrCl···I$^-$	2.541	0.368	2.665	0.313	–32.6	–32.8	14.8	–47.6
ICl···F$^-$	2.637	0.285	1.955	0.291	–50.3	–50.7	8.7	–59.4
ICl···Cl$^-$	2.654	0.302	2.399	0.376	–31.9	–32.2	9.5	–41.7
ICl···Br$^-$	2.665	0.313	2.541	0.368	–29.3	–29.5	10.1	–39.6
ICl···I$^-$	2.700	0.348	2.700	0.348	–28.6	–28.8	11.9	–40.7

[a] Computed at ZORA-BP86/TZ2P. [b] D–X stretch relative to optimized DX molecule. [c] X···A$^-$ distance relative to optimized XA molecule.

Table S4. Bond lengths (in Å) and energies (in kcal mol^{-1}) relative to reactants of bromine-bonded complexes.[a]

DX···A$^-$	$r_{D\text{-}X}$	$\Delta r_{D\text{-}X}$[b]	$r_{X\cdots A^-}$	$\Delta r_{X\cdots A^-}$[c]	ΔH^{298}	ΔE	ΔE_{strain}	ΔE_{int}
FBr···F$^-$	2.009	0.213	2.009	0.213	–70.5	–70.9	8.6	–79.5
FBr···Cl$^-$	2.018	0.222	2.454	0.281	–48.5	–48.7	9.2	–57.8
FBr···Br$^-$	2.023	0.227	2.601	0.280	–44.5	–44.7	9.5	–54.2
FBr···I$^-$	2.036	0.240	2.775	0.269	–42.3	–42.4	10.4	–52.9
ClBr···F$^-$	2.454	0.281	2.018	0.222	–65.3	–65.7	9.7	–75.5
ClBr···Cl$^-$	2.465	0.292	2.465	0.292	–43.7	–44.0	10.4	–54.3
ClBr···Br$^-$	2.473	0.300	2.612	0.291	–40.0	–40.2	10.8	–51.0
ClBr···I$^-$	2.495	0.322	2.786	0.280	–38.2	–38.5	12.1	–50.6
BrBr···F$^-$	2.601	0.280	2.023	0.227	–63.8	–64.3	8.6	–72.9
BrBr···Cl$^-$	2.612	0.291	2.473	0.300	–42.5	–42.8	9.2	–52.1
BrBr···Br$^-$	2.621	0.300	2.621	0.300	–39.0	–39.2	9.7	–48.9
BrBr···I$^-$	2.644	0.323	2.794	0.288	–37.4	–37.7	10.9	–48.6
IBr···F$^-$	2.775	0.269	2.036	0.240	–59.4	–59.8	7.1	–67.0
IBr···Cl$^-$	2.786	0.280	2.495	0.322	–38.5	–38.8	7.6	–46.4
IBr···Br$^-$	2.794	0.288	2.644	0.323	–35.2	–35.5	7.9	–43.4
IBr···I$^-$	2.818	0.312	2.818	0.312	–33.8	–34.0	9.0	–43.1

[a] Computed at ZORA-BP86/TZ2P. [b] D–X stretch relative to optimized DX molecule. [c] X···A$^-$ distance relative to optimized XA molecule.

Table S5. Bond lengths (in Å) and energies (in kcal mol^{-1}) relative to reactants of iodine-bonded complexes.[a]

DX···A$^-$	$r_{\text{D-X}}$	$\Delta r_{\text{D-X}}$[b]	$r_{\text{X}\cdots\text{A}^-}$	$\Delta r_{\text{X}\cdots\text{A}^-}$[c]	ΔH^{298}	ΔE	ΔE_{strain}	ΔE_{int}
FI···F$^-$	2.129	0.184	2.129	0.184	–74.6	–75.0	6.1	–81.1
FI···Cl$^-$	2.124	0.179	2.620	0.268	–49.6	–49.8	5.8	–55.6
FI···Br$^-$	2.126	0.181	2.781	0.275	–44.9	–45.1	5.9	–51.0
FI···I$^-$	2.132	0.187	2.977	0.277	–41.8	–41.9	6.3	–48.2
ClI···F$^-$	2.620	0.268	2.124	0.179	–72.8	–73.3	7.8	–81.1
ClI···Cl$^-$	2.615	0.263	2.615	0.263	–47.7	–48.0	7.6	–55.6
ClI···Br$^-$	2.620	0.268	2.776	0.270	–43.2	–43.5	7.8	–51.3
ClI···I$^-$	2.632	0.280	2.971	0.271	–40.4	–40.6	8.4	–49.0
BrI···F$^-$	2.781	0.275	2.126	0.181	–71.8	–72.2	7.4	–79.6
BrI···Cl$^-$	2.776	0.270	2.620	0.268	–46.8	–47.1	7.2	–54.3
BrI···Br$^-$	2.782	0.276	2.782	0.276	–42.4	–42.7	7.4	–50.1
BrI···I$^-$	2.795	0.289	2.976	0.276	–39.7	–40.0	8.0	–48.0
II···F$^-$	2.977	0.277	2.132	0.187	–68.5	–69.0	6.4	–75.4
II···Cl$^-$	2.971	0.271	2.632	0.280	–43.8	–44.2	6.1	–50.3
II···Br$^-$	2.976	0.276	2.795	0.289	–39.6	–39.9	6.4	–46.3
II···I$^-$	2.991	0.291	2.991	0.291	–37.1	–37.4	6.9	–44.3

[a] Computed at ZORA-BP86/TZ2P. [b] D–X stretch relative to optimized DX molecule. [c] X···A$^-$ distance relative to optimized XA molecule.

Table S6. Analysis of hydrogen-bonding mechanism in DH···A$^-$ complexes.[a]

DX···A$^-$	ΔE_{int}	ΔV_{elstat}	ΔE_{Pauli}	ΔE_{oi}	ΔE^{σ}_{oi}	ΔE^{π}_{oi}	$\langle \sigma^* \mid np \rangle$	σ^* pop.	np pop.	$Q_{A^-}^{VDD}$
FH···F$^-$	–72.8	–76.4	68.8	–65.1	–58.1	–7.0	0.268	0.27	1.76	–0.51
FH···Cl$^-$	–29.8	–31.9	24.3	–22.2	–20.0	–2.2	0.359	0.14	1.84	–0.66
FH···Br$^-$	–23.9	–25.4	18.5	–17.0	–15.4	–1.6	0.390	0.11	1.88	–0.68
FH···I$^-$	–19.4	–20.2	14.5	–13.7	–12.6	–1.2	0.421	0.13	1.89	–0.69
ClH···F$^-$	–111.9	–98.2	124.9	–138.7	–128.2	–10.4	0.282	0.54	1.67	–0.36
ClH···Cl$^-$	–47.2	–45.2	65.4	–67.3	–62.7	–4.7	0.341	0.41	1.63	–0.48
ClH···Br$^-$	–30.9	–31.5	41.7	–41.1	–38.2	–2.9	0.346	0.30	1.72	–0.56
ClH···I$^-$	–22.1	–22.6	28.1	–27.6	–25.6	–1.9	0.357	0.27	1.77	–0.62
BrH···F$^-$	–118.8	–101.8	138.8	–155.9	–144.9	–10.9	0.276	0.60	1.66	–0.33
BrH···Cl$^-$	–61.5	–54.4	91.9	–99.0	–92.7	–6.3	0.337	0.55	1.53	–0.38
BrH···Br$^-$	–41.3	–39.2	64.0	–66.2	–62.2	–4.0	0.336	0.44	1.60	–0.46
BrH···I$^-$	–28.4	–27.1	42.4	–43.7	–41.1	–2.6	0.338	0.39	1.66	–0.53
IH···F$^-$	–121.4	–104.6	156.9	–173.7	–163.0	–10.7	0.264	0.66	1.65	–0.31
IH···Cl$^-$	–69.6	–62.1	116.1	–123.6	–116.6	–7.1	0.324	0.66	1.46	–0.32
IH···Br$^-$	–50.3	–47.8	89.6	–92.1	–87.3	–4.8	0.320	0.57	1.49	–0.38
IH···I$^-$	–35.4	–33.8	63.0	–64.6	–61.3	–3.2	0.315	0.52	1.55	–0.45

[a] Computed at ZORA-BP86/TZ2P: Decomposition of ΔE_{int} (in kcal mol^{-1}), see also eq. 2; $\langle \sigma^* \mid np \rangle$ = overlap of antibonding σ^* acceptor orbital on DX fragment with σ lone-pair np orbital on halide A$^-$; pop. = population (in electrons) of indicated orbital; $Q_{A^-}^{VDD}$ = VDD charge on halide A$^-$ (in a.u.).

Table S7. Analysis of fluorine-bonding mechanism in DF···A⁻ complexes.[a]

DX···A⁻	ΔE_{int}	ΔV_{elstat}	ΔE_{Pauli}	ΔE_{oi}	ΔE^{σ}_{oi}	ΔE^{π}_{oi}	$\langle \sigma^* \mid np \rangle$	σ^* pop.	np pop.	$Q_{A^-}^{VDD}$
FF···F⁻	–75.0	–41.0	73.2	–107.1	–106.2	–1.0	0.125	0.59	1.43	–0.42
FF···Cl⁻	–77.5	–52.4	107.8	–132.9	–128.7	–4.2	0.146	0.76	1.26	–0.33
FF···Br⁻	–81.7	–53.8	111.7	–139.6	–134.8	–4.8	0.145	0.82	1.20	–0.30
FF···I⁻	–94.3	–61.7	132.2	–164.8	–158.0	–6.7	0.145	0.95	1.05	–0.27
ClF···F⁻	–46.7	–17.3	51.7	–81.2	–81.2	0.0	0.107	0.54	1.49	–0.48
ClF···Cl⁻	–47.3	–30.2	78.3	–95.4	–93.6	–1.7	0.133	0.68	1.35	–0.38
ClF···Br⁻	–53.6	–37.6	91.8	–107.8	–105.2	–2.6	0.139	0.77	1.26	–0.33
ClF···I⁻	–70.4	–53.4	124.6	–141.6	–136.5	–5.1	0.144	0.95	1.08	–0.27
BrF···F⁻	–40.6	–11.0	45.9	–75.5	–75.5	–0.1	0.099	0.52	1.51	–0.49
BrF···Cl⁻	–37.2	–21.4	64.3	–80.0	–78.9	–1.1	0.124	0.63	1.40	–0.41
BrF···Br⁻	–42.3	–29.1	77.6	–90.9	–89.1	–1.8	0.131	0.71	1.32	–0.37
BrF···I⁻	–57.9	–45.9	111.9	–123.8	–119.8	–4.1	0.140	0.89	1.14	–0.29
IF···F⁻	–29.8	–1.8	33.7	–61.8	–61.4	–0.4	0.087	0.48	1.55	–0.53
IF···Cl⁻	–22.3	–8.3	40.3	–54.4	–53.7	–0.7	0.108	0.53	1.50	–0.49
IF···Br⁻	–25.0	–14.6	50.3	–60.7	–59.8	–0.9	0.118	0.59	1.45	–0.44
IF···I⁻	–36.2	–29.6	79.8	–86.5	–84.4	–2.1	0.133	0.75	1.29	–0.36

[a] Computed at ZORA-BP86/TZ2P: Decomposition of ΔE_{int} (in kcal mol^{-1}), see also eq. 2; $\langle \sigma^* \mid np \rangle$ = overlap of antibonding σ^* acceptor orbital on DX fragment with σ lone-pair np orbital on halide A⁻; pop. = population (in electrons) of indicated orbital; $Q_{A^-}^{VDD}$ = VDD charge on halide A⁻ (in a.u.).

Table S8. Analysis of chlorine-bonding mechanism in DCl···A^- complexes.[a]

DX···A^-	ΔE_{int}	ΔV_{elstat}	ΔE_{Pauli}	ΔE_{oi}	ΔE^{σ}_{oi}	ΔE^{π}_{oi}	$\langle \sigma^* \mid np \rangle$	σ^* pop.	n*p* pop.	$Q_{A^-}^{VDD}$
FCl···F^-	–76.4	–85.5	107.2	–98.1	–91.1	–7.0	0.187	0.45	1.57	–0.45
FCl···Cl^-	–57.1	–61.6	86.4	–81.9	–76.5	–5.4	0.209	0.53	1.50	–0.46
FCl···Br^-	–54.3	–57.3	82.1	–79.1	–74.3	–4.8	0.207	0.54	1.49	–0.45
FCl···I^-	–54.3	–54.5	81.8	–81.6	–76.8	–4.8	0.206	0.61	1.41	–0.42
ClCl···F^-	–70.0	–74.8	109.3	–104.4	–98.8	–5.6	0.167	0.51	1.55	–0.43
ClCl···Cl^-	–51.6	–54.1	86.2	–83.7	–79.3	–4.3	0.190	0.58	1.46	–0.43
ClCl···Br^-	–49.3	–50.8	81.2	–79.7	–75.9	–3.8	0.191	0.60	1.44	–0.42
ClCl···I^-	–50.5	–49.7	81.7	–82.5	–78.6	–3.9	0.192	0.67	1.35	–0.39
BrCl···F^-	–66.6	–70.1	108.8	–105.3	–100.1	–5.3	0.160	0.52	1.54	–0.43
BrCl···Cl^-	–48.6	–50.5	84.9	–83.1	–79.1	–4.0	0.182	0.59	1.46	–0.42
BrCl···Br^-	–46.4	–47.6	79.9	–78.8	–75.3	–3.5	0.183	0.61	1.43	–0.42
BrCl···I^-	–47.6	–46.9	80.6	–81.3	–77.7	–3.7	0.185	0.68	1.35	–0.38
ICl···F^-	–59.4	–61.3	104.9	–103.0	–98.5	–4.5	0.148	0.53	1.55	–0.43
ICl···Cl^-	–41.7	–43.1	79.1	–77.8	–74.5	–3.2	0.170	0.58	1.47	–0.44
ICl···Br^-	–39.6	–40.9	74.4	–73.1	–70.3	–2.7	0.172	0.60	1.45	–0.43
ICl···I^-	–40.7	–40.9	75.1	–75.0	–72.1	–2.9	0.175	0.67	1.37	–0.39

[a] Computed at ZORA-BP86/TZ2P: Decomposition of ΔE_{int} (in kcal mol^{-1}), see also eq. 2; $\langle \sigma^* \mid np \rangle$ = overlap of anti-bonding σ^* acceptor orbital on DX fragment with σ lone-pair n*p* orbital on halide A^-; pop. = population (in electrons) of indicated orbital; $Q_{A^-}^{VDD}$ = VDD charge on halide A^- (in a.u.).

Table S9. Analysis of bromine-bonding mechanism in DBr···A⁻ complexes.[a]

DX···A⁻	ΔE_{int}	ΔV_{elstat}	ΔE_{Pauli}	ΔE_{oi}	$\Delta E^{\sigma}_{\text{oi}}$	$\Delta E^{\pi}_{\text{oi}}$	$\langle \sigma^* \mid np \rangle$	σ^* pop.	n*p* pop.	$Q_{\text{A}^-}^{\text{VDD}}$
FBr···F⁻	–79.5	–92.3	98.8	–86.0	–77.7	–8.2	0.201	0.43	1.60	–0.45
FBr···Cl⁻	–57.8	–65.9	79.1	–71.0	–65.6	–5.5	0.234	0.51	1.50	–0.47
FBr···Br⁻	–54.2	–61.5	75.6	–68.3	–63.6	–4.7	0.235	0.52	1.49	–0.46
FBr···I⁻	–52.9	–57.9	74.8	–69.8	–65.3	–4.4	0.235	0.58	1.43	–0.44
ClBr···F⁻	–75.5	–84.6	103.1	–94.0	–86.9	–7.1	0.181	0.48	1.58	–0.43
ClBr···Cl⁻	–54.3	–60.5	81.1	–75.0	–70.2	–4.8	0.212	0.55	1.48	–0.44
ClBr···Br⁻	–51.0	–56.8	77.1	–71.3	–67.2	–4.1	0.214	0.57	1.45	–0.44
ClBr···I⁻	–50.6	–54.3	76.3	–72.5	–68.6	–3.9	0.216	0.64	1.38	–0.41
BrBr···F⁻	–72.9	–80.8	103.9	–96.0	–89.3	–6.7	0.173	0.49	1.57	–0.43
BrBr···Cl⁻	–52.1	–57.6	81.1	–75.5	–70.9	–4.5	0.204	0.56	1.47	–0.44
BrBr···Br⁻	–48.9	–54.1	76.6	–71.4	–67.6	–3.9	0.206	0.58	1.45	–0.43
BrBr···I⁻	–48.6	–52.0	76.0	–72.5	–68.7	–3.8	0.208	0.65	1.36	–0.40
IBr···F⁻	–67.0	–74.0	103.2	–96.2	–90.1	–6.1	0.162	0.50	1.57	–0.43
IBr···Cl⁻	–46.4	–51.8	78.3	–73.0	–69.0	–3.9	0.192	0.56	1.48	–0.44
IBr···Br⁻	–43.4	–48.9	73.9	–68.5	–65.1	–3.3	0.194	0.57	1.46	–0.43
IBr···I⁻	–43.1	–47.2	73.1	–68.9	–65.7	–3.2	0.197	0.64	1.39	–0.40

[a] Computed at ZORA-BP86/TZ2P: Decomposition of ΔE_{int} (in kcal mol^{-1}), see also eq. 2; $\langle \sigma^* \mid np \rangle$ = overlap of antibonding σ^* acceptor orbital on DX fragment with σ lone-pair n*p* orbital on halide A⁻; pop. = population (in electrons) of indicated orbital; $Q_{\text{A}^-}^{\text{VDD}}$ = VDD charge on halide A⁻ (in a.u.).

Table S10. Analysis of iondine-bonding mechanism in DI···A⁻ complexes.[a]

DX···A⁻	ΔE_{int}	ΔV_{elstat}	ΔE_{Pauli}	ΔE_{oi}	ΔE^{σ}_{oi}	ΔE^{π}_{oi}	$\langle \sigma^* \mid np \rangle$	σ^* pop.	np pop.	$Q_{A^-}^{VDD}$
FI···F⁻	–81.1	–103.5	100.0	–77.7	–66.8	–10.9	0.205	0.36	1.66	–0.44
FI···Cl⁻	–55.6	–69.1	73.8	–60.3	–54.0	–6.3	0.252	0.44	1.57	–0.49
FI···Br⁻	–51.0	–63.7	69.8	–57.0	–51.8	–5.2	0.255	0.45	1.56	–0.49
FI···I⁻	–48.2	–58.5	67.1	–56.8	–52.2	–4.6	0.258	0.51	1.51	–0.48
ClI···F⁻	–81.1	–101.2	107.6	–87.5	–77.5	–10.0	0.190	0.42	1.64	–0.41
ClI···Cl⁻	–55.6	–67.5	78.6	–66.7	–60.9	–5.9	0.232	0.49	1.54	–0.46
ClI···Br⁻	–51.3	–62.4	74.0	–62.8	–57.9	–5.0	0.235	0.50	1.52	–0.46
ClI···I⁻	–49.0	–57.6	71.0	–62.4	–58.0	–4.4	0.237	0.56	1.46	–0.44
BrI···F⁻	–79.6	–98.7	109.6	–90.5	–80.9	–9.6	0.183	0.44	1.64	–0.41
BrI···Cl⁻	–54.3	–65.4	79.3	–68.2	–62.5	–5.7	0.223	0.50	1.53	–0.45
BrI···Br⁻	–50.1	–60.5	74.4	–64.0	–59.2	–4.8	0.226	0.52	1.51	–0.45
BrI···I⁻	–48.0	–56.0	71.3	–63.3	–59.0	–4.3	0.229	0.58	1.54	–0.43
II···F⁻	–75.4	–94.0	111.6	–92.9	–83.8	–9.1	0.173	0.45	1.63	–0.40
II···Cl⁻	–50.3	–61.3	79.1	–68.1	–62.9	–5.2	0.211	0.51	1.53	–0.45
II···Br⁻	–46.3	–56.8	73.9	–63.4	–59.0	–4.4	0.214	0.52	1.51	–0.45
II···I⁻	–44.3	–52.6	70.6	–62.3	–58.4	–3.9	0.217	0.58	1.46	–0.43

[a] Computed at ZORA-BP86/TZ2P: Decomposition of ΔE_{int} (in kcal mol^{-1}), see also eq. 2; $\langle \sigma^* \mid np \rangle$ = overlap of antibonding σ^* acceptor orbital on DX fragment with σ lone-pair np orbital on halide A⁻; pop. = population (in electrons) of indicated orbital; $Q_{A^-}^{VDD}$ = VDD charge on halide A⁻ (in a.u.).
